# A systematic review of health economic evaluation quality assessment instruments for medical devices

**DOI:** 10.1017/S0266462325000212

**Published:** 2025-07-10

**Authors:** Ilke Akpinar, Ali Unsal, Mike Paulden, Jeff Round

**Affiliations:** 1 College of Health Sciences, School of Public Health, University of Alberta, Edmonton, AB, Canada; 2 Institute of Health Economics, Industry Partnership, Edmonton, AB, Canada; 3 Faculty of Medicine and Dentistry, Pediatrics Department, University of Alberta, Edmonton, AB, Canada

**Keywords:** medical device, economic evaluation, quality assessment, checklist, methodological quality

## Abstract

**Objectives:**

Health economic evaluations are important for healthcare resource allocation. Reviews of health economic evaluations for medical devices have highlighted concerns about the quality of these studies. The complexity of medical devices, including learning curve effects, organizational impact, dynamic pricing, low evidence, and incremental innovation presents unique challenges compared with pharmaceuticals. To support developing a methodological quality assessment instrument for medical device economic evaluations, we conducted a systematic review to identify and evaluate existing economic evaluation quality assessment instruments for suitability in medical device evaluations.

**Methods:**

A comprehensive search of databases (MEDLINE, EMBASE, EconLit, CINAHL, and Web of Science) and grey literature was conducted. Two reviewers screened titles and abstracts. Full-text, peer-reviewed primary studies introducing original instruments were included. Only methodological quality assessment instruments were considered for data extraction. Each item was assessed for its suitability in evaluating medical device economic evaluations and inclusion of medical device-specific features.

**Results:**

The search identified 4203 citations and 77 grey literature sources. Fifteen results underwent full-text assessment, with five relevant instruments identified. A previous systematic review identified 10 additional instruments, which we also considered. Of these 25 articles, 13 were included in the review. These instruments lack specificity for medical devices, particularly in addressing features like learning curve effects, organizational impact, and incremental innovation. Instruments should include items specific to these unique characteristics.

**Conclusions:**

Existing instruments contain general items related to health economic evaluation studies, highlighting the need for an instrument specifically tailored to evaluate the methodological quality of medical device economic evaluation studies.

## Background

Health economic evaluations are valuable tools for guiding policymakers in allocating scarce healthcare resources. Quality assessment is important for maintaining methodological standards to obtain valid and reliable results (1). It enhances study transparency and reproducibility and facilitates appropriate resource allocation within healthcare systems (2). Reviews conducted since 2015 on health economic evaluations for medical devices have highlighted concerns about the quality of these studies (3;4) indicating that they are often insufficient to address the important features of medical devices (5;6). The value, accessibility, and affordability of new medical devices are critical considerations for patients, healthcare providers, and health systems, alongside their effectiveness and safety. The cost-effectiveness of these technologies and the most appropriate ways to evaluate them are of increasing importance (5).

The economic evaluation of medical devices differs from pharmaceuticals in several important ways (7–10). Key considerations include limited clinical and economic evidence (5;11;12), learning curve effects (5;7), organizational impact (5;7;11), incremental innovation (5;7;10;13;14), dynamic pricing (15;16), diversity in device types and applications (14), and challenges with transferability of results (14). ‘Insufficient evidence’ in evaluating medical devices refers to the limitations of randomized clinical trials, including lack of randomization, small sample sizes, and short follow-up periods. These limitations make it difficult to draw definitive conclusions about the effectiveness and cost-effectiveness of devices in real-world settings (5;11;12). The ‘learning curve’ describes the improvement in user proficiency over time (17). The ‘organizational impact’ of a medical device includes various factors affecting its adoption, use, and integration within the healthcare system, with user education and organizational adjustments being essential for maximizing its benefits (5). ‘Incremental innovation’ in medical devices refers to the continuous improvements and modifications made over the device’s lifecycle (7;10;13;14). ‘Dynamic pricing’ in the context of medical devices refers to the fluctuating costs associated with new devices and their consumables, influenced by factors such as market monopolies, manufacturer pricing strategies, and ongoing incremental innovations (7;11;14). ‘Diversity’ in medical devices refers to the range of differences in complexity, features, usability, technological specifications, and clinical settings (14). ‘Transferability’ in medical device economic evaluations refers to the challenge of applying cost-effectiveness results across different healthcare settings, often complicated by variations in device features, clinical usage, and additional cost components, all of which increase uncertainty (14).

A more rigorous approach is necessary to explore the impact of these various aspects; however, to our knowledge, there is currently no methodological quality assessment instrument designed specifically for medical device economic evaluations. To qualify as ‘specifically tailored’ for medical devices, an instrument should incorporate criteria enabling the assessment of one or more of the seven defined features essential to their evaluation, or alternatively, contain items that adequately address these features.

In 2012, the Agency for Healthcare Research and Quality (AHRQ) in the United States conducted a systematic literature review to assess the best practices for conducting and reporting health economic evaluations (18). Ten quality assessment instruments (19–28) published between 1992 and 2011 were identified. To identify additional instruments, including those published after 2012 and in grey literature sources, we conducted a systematic literature review of methodological quality assessment instruments for medical device economic evaluations. This review aims to capture recent advancements and address specific considerations for medical device economic evaluations, which were not thoroughly covered in the prior review.

Our primary aim was to identify, summarize, and assess the relevance of existing instruments for evaluating medical device economic evaluations, focusing on seven defined medical device-specific features. This review has two key objectives: (i) to determine whether any existing quality assessment instruments are specifically tailored for medical device economic evaluations, and (ii) in the absence of a suitable instrument, to evaluate each item within current methodological quality assessment instruments for its potential inclusion. These items will be assessed based on their relevance to the seven device-specific features and included in a Delphi pool for expert consensus in the next phase of this project.

## Methods

The reporting of this systematic review was guided by the standards of the Preferred Reporting Items for Systematic Review and Meta-Analysis (PRISMA) Statement (29). The PRISMA checklists are available in Supplementary Materials 2 and 3. Our protocol, “Health Economic Evaluation Methodological Quality Assessment Tools: A protocol for a systematic review,” was registered with the International Platform of Registered Systematic Review and Meta-analysis Protocols (INPLASY DOI: 10.37766/inplasy2023.7.0093).

### Eligibility criteria

Eligible studies were full-text, peer-reviewed primary studies introducing original instruments designed to assess economic evaluations of medical devices. Updated versions of instruments offering a different perspective were also included.

We excluded studies that focus on frameworks or guidelines for conducting economic evaluations, as well as those that adopt an original tool or checklist for purposes other than medical device economic evaluation, or that describe or validate an existing instrument. Reviews (scoping, rapid, systematic, literature), editorials, commentary, conference abstracts, dissertations, and these were also excluded. Studies published in a language other than English were excluded.

### Information sources

We searched electronic databases—Ovid MEDLINE, Ovid EMBASE, CINAHL (via EBSCOhost), EconLit (via EBSCOhost), and Web of Science (via its online interface)—for English-language literature published between January 1, 2012 and May 24, 2023, with the final search conducted on May 24, 2023. A grey literature search was performed between November 16 and November 30, 2023, using the CADTH Grey Matters tool (30), as well as the International Network of Agencies for Health Technology Assessment (INAHTA) database and the Professional Society for Health Economics and Outcomes Research (ISPOR) website, to locate relevant guidance documents and instruments.

### Search strategy

A systematic review search strategy was designed in collaboration with a University of Alberta Health Sciences librarian experienced in systematic reviews. We used the previous systematic review (18) search strategy as a foundation and made several adjustments to improve its relevance to our study. While the previous review used terms like “cost–benefit analysis,” “cost of illness,” and “economic evaluation” to capture economic analyses, we expanded the scope to specifically identify studies on quality assessment tools. To achieve this, we introduced terms such as “checklist,” “tool,” “questionnaire,” and included names of widely used checklists. Search terms included a combination of controlled vocabulary (e.g., Medical Subject Headings and EMBASE Subject Headings) and relevant keywords related to medical device economic evaluations. Additionally, we reviewed reference lists of included articles to identify further studies. The full electronic search strategy, including all limits and filters applied, is provided in the Supplementary Material 1.

### Selection process

The results of the initial searches were downloaded into EndNote (31) reference manager. Duplicate articles retrieved from multiple databases were removed, and the remaining articles were uploaded to Covidence (32), a web-based systematic review manager. Covidence was used to track the search results throughout the title and abstract review, article selection, and data extraction stages.

Titles and abstracts of all citations identified in the searches were screened in duplicate (IA, AU) to assess potential relevancy. The full text of any potentially relevant articles was also assessed in duplicate against the selection criteria. Discrepancies were resolved by consensus, with a third reviewer (MP) providing arbitration, as necessary. Tool or checklist eligibility was defined based on the definition by Zoratti et al. (33). Reporting checklists were defined as “instruments that are used to evaluate the presence or absence of components without value on that component’s use.” Critical appraisal tools were defined as “an extension of reporting checklists and include some interpretation or evaluation of the reported content.” (33)

While the relevance of methods such as sensitivity analyses, risk of bias assessment for missing results, and assessing certainty in evidence for many systematic reviews, these methods were not directly applicable to our synthesis of methodological quality assessment tools. Our review focuses on evaluating and synthesizing existing methodological quality assessment tools to assess their effectiveness and applicability in medical device economic evaluations, rather than synthesizing quantitative outcomes. As a result, methods such as sensitivity analyses and bias assessments for missing data were not relevant in this context. Additionally, assessing certainty in the evidence is more suited to clinical or outcome-based reviews, rather than methodological reviews. Instead, we focused on ensuring methodological rigor in study selection and maintaining transparency throughout the synthesis process.

### Data collection process

One reviewer (IA) extracted the data from each article to a data extraction form developed by IA. A second reviewer (AU) cross-checked all extracted data for accuracy and consistency. Data discrepancies within articles were noted and it was established that data extraction was prioritized to come from the summary of tables, supplemented by the main text as needed.

### Data items

The following data elements were extracted:

- Descriptive characteristics of the published instruments (e.g., name, first author, year of publication, author affiliation, journal, number of items, item response options, intended use, target audience, the methods of development, funding source, any validation data)
- Only from methodological quality assessment instruments:
  - Each item and its appropriateness to assess medical device economic evaluations.
  - Content review with respect to the seven medical device-specific features (insufficient evidence, learning curve effects, organizational impact, incremental innovation, dynamic pricing, diversity, and transferability of the results).

## Results

### Study selection

**Figure 1.**
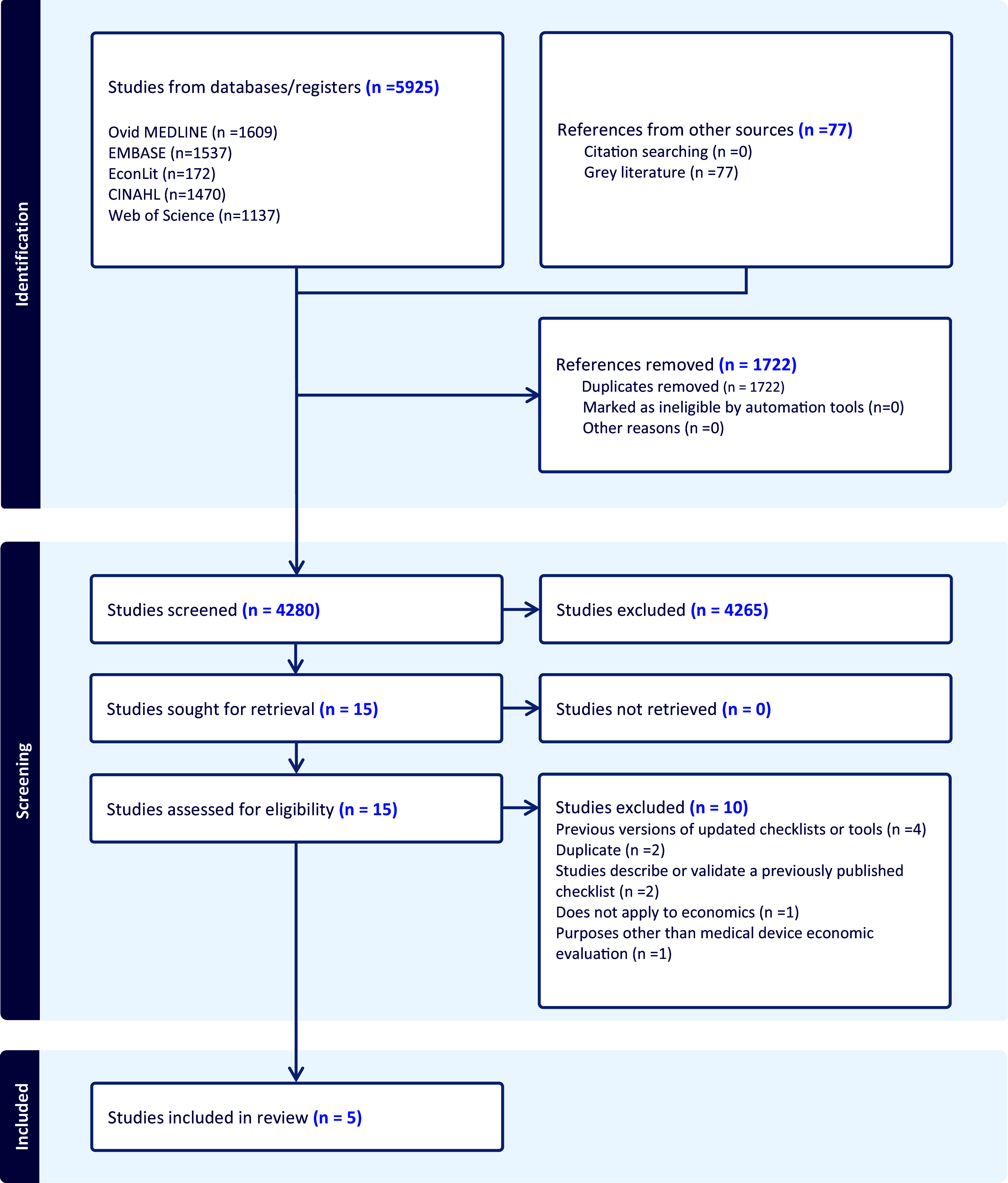
PRISMA flow diagram. Source: Page MJ, et al. BMJ 2021;372:n71. doi: 10.1136/bmj.n71. This work is licensed under CC BY 4.0. To view a copy of this license, visit https://creativecommons.org/licenses/by/4.0/

In the initial electronic literature search, 6,002 records were identified. After removing duplicates, a total of 4,280 records remained. Screening of 4,203 titles and abstracts and 77 grey literature sources led to the retrieval of 15 articles for full-text review. The 2012 review from Walker et al. (18) identified 10 instruments that we considered as well. Of these 25 articles, 15 were deemed eligible and included in the review (2;19–28;34–37). The PRISMA (Preferred Reporting Items for Systematic Reviews and Meta-Analyses) study flowchart detailing the process of study selection and exclusion is provided in Figure 1.

After a full-text review, two instruments (2;37) were excluded because they were specifically designed as reporting quality assessment instruments.

### Study characteristics

Thirteen instruments (19–28;34–36) were designed for the methodological quality assessment of health economic evaluations in general, rather than with a specific focus on medical device economic evaluations. Detailed characteristics of each instrument are summarized in Table 1.

**Table 1.** Included quality assessment instruments

Instrument/first author/publication year	Number of items	Item response options	Intended use	Target audience	Methods of development
***Economic Evaluation Scoring Worksheet*** Adams et al. (23)	29	Varies	To make recommendations for performing adequate economic analyses if investigators plan to include them in future clinical trials	Investigators using economic analyses in clinical trials	Not clear (based on a textbook)
***CUA checklist*** Gerard et al. (24)	37	Open ended	To develop the evaluation criteria for CUAs	Policymakers and researchers who use CUAs	The criteria used by Drummond et al. were used as reference point Reviewed by 18 international researchers
***Checklist to evaluate pharmacoeconomic studies*** Sacristan et al. (25)	39	Correct Acceptable Doubtful Incorrect or NA	Guidance	Researchers, journal editors, and audiences	Analyzing the most relevant studies on the subject
***Methodological and conduct principles for pharmacoeconomic research*** Clemens et al. (26)	21	Open ended	Guidance	Pharmaceutical industry	The principles were reviewed by a panel of academic experts and outside reviewers at each stage of development
***Checklist for reporting the reference case cost effectiveness analysis*** US Panel (27)	38	Open ended	To improve the quality and accessibility of CEA reports	Not explicitly stated	The panel reviewed the theoretical foundations of CEA, current practices, and alternative methods used in analyses Recommendations were developed
***BMJ checklist*** Drummond et al. (21)	35	Yes No Not clear Not appropriate	Guidance	Specialists, non-specialist readers of economic work; authors; editors	Broad consensus (Drafts of the checklist were circulated to health economists and journal editors, at the biannual meeting of the UK Health Economists’ Study Group)
***The pediatric quality appraisal questionnaire*** Ungar et al. (22)	57	Yes (explicitly stated) Yes (inferring from text, tables, or figures) No NA	To develop a pediatric-specific quality appraisal instrument	Health economists, methods researchers, and policy decision-makers	A draft instrument was constructed from published checklists New questions pertaining to the pediatric population were incorporated An expert panel reviewed the draft instrument and the proposed scoring scheme for face and content validity
***The quality of health economic studies instrument*** Chiou et al. (19)	16 (weighted)	Yes No Full value or zero	To provide a practical quantitative tool for appraising the quality of cost-effectiveness studies	Decision-makers, researchers, editors/reviewers	Collected data to develop weights for each criterion Collected data to validate the quantitative, weighted instrument against the global quality assessment of experts reviewing actual economic articles
***Criteria list for assessment of methodological*** ***quality of economic evaluations*** Evers et al. (20)	19	Yes No	To develop a criteria list for assessment of the methodological quality of economic evaluations in systematic reviews	Healthcare professionals, consumers, researchers, and policy-makers	Three Delphi rounds (23 international experts)
***Checklist to frame health technology assessments*** Grutters et al. (28)	11	Open ended	To develop and apply a checklist to systematically frame HTAs in a way that they are applicable to the decision problem assess if a decision problem can be informed by an available HTA	Decisionmakers	Literature review Collaboration with clinicians and policymakers
***Checklist for economic evaluations of pharmaceuticals*** Inotai et al. (35)	91	Yes No Not relevant	To develop a scientifically rigorous and detailed appraisal checklist for economic evaluations of pharmaceuticals in the single health technology assessment process	Researchers, decision-makers	Development: Expert panel (2 academic experts) Approval: members of the HTA Office
***AGREEDT Checklist*** Kip et al. (37)	44	Yes No Requires an explanation	To design a reporting checklist for model-based health economic evaluations of diagnostic tests and biomarkers	Researchers, decision-makers	Scoping review Critical review by the 4 experts
***TRUST tool*** Grimm et al. (34)	The rows (10) present model aspects, and columns (5) relate to sources of uncertainty	Yes Unknown Likely high Likely low Likely no impact	To assess uncertainty in health economic models	Researchers, decision-makers	HTA stakeholder discussion meetings and interviews
***CHEERS 2022*** Husereau et al. (2)	28	The section of the manuscript where relevant information can be found Not applicable Not reported	To assess reporting quality, user guide	Researchers, peer reviewers, editors	Delphi Panel exercise
***CHEQUE tool*** Kim et al. (36)	48	Yes Somewhat No NA Scoring weight assessment	To assess the quality of CEAs, separately for methods and reporting quality	Decision makers, researchers, and practitioners	The best-worst scaling survey

AGREEDT: Alignment in the Reporting of Economic Evaluations of Diagnostic Tests and Biomarkers; BMJ: British Medical Journal; CEA: cost-effectiveness analysis; CHEERS 2022: Consolidated Health Economic Evaluation Reporting Standards 2022; CHEQUE: Criteria for Health Economic Quality Evaluation; CUA: cost-utility analysis; NA: not applicable; TRUST: Transparent Uncertainty Assessment.

The included studies encompass a variety of instruments designed to assess health economic evaluations. These instruments, developed between 1992 and 2023, vary in their number of items, with some containing as few as 16 (19) and others as many as 91 (35). The item response options also vary, including open-ended responses (24–28), yes/no options (19;35–37), and weighted scales (19). Intended uses range from providing guidance for economic analyses in clinical trials (23) to developing evaluation criteria for cost-utility analyses (24) and assessing the quality of cost-effectiveness studies (19). The target audiences for quality assessment checklists include researchers (19;20;22–25;34–36), decision-makers (19;22;28;34–36), policy-makers (20;22;24), journal editors (19;21;25) and pharmaceutical industry professionals (26). One checklist’s authors (27) did not explicitly state the target audience. The development methods of these instruments vary, including literature reviews, expert panel reviews, and collaboration with clinicians and policymakers.

Table 2 presents potentially relevant items extracted from various instruments, providing a comprehensive overview of key considerations in medical device economic evaluations. Out of 388 items from 13 methodological quality assessment instruments, only seven items, found in four instruments (23;24;28;35), were relevant to medical device economic evaluations. Relevance was assessed by comparing each checklist item against specific criteria developed for medical device economic evaluations, including the seven items outlined in the background section. These include considerations for dynamic pricing (23;35), low evidence cases (35), learning curve effects (28), incremental innovation (24), and organizational impact (35). This approach ensured a focus on aspects important to medical devices. None of the identified instruments in the review were deemed suitable for the standalone evaluation of medical devices.

**Table 2.** Medical device-specific features and relevant items by instrument

Medical device specific feature	Quality assessment instrument	Relevant item
**Dynamic pricing**	***Economic evaluation scoring worksheet*** Adams et al. (23)	Was depreciation considered appropriately and measured correctly?
	***Checklist for economic evaluations of pharmaceuticals*** Inotai et al. (35)	Were the expected sales of the investigated technology appropriately estimated?
**Low evidence**	***Checklist for economic evaluations of pharmaceuticals*** Inotai et al. (35)	In case of statistically not significant non-inferiority, is the new technology expected to result in at least the same health benefit as the comparator? (that is if clinical trials had been many times larger, would the presumable conclusion still be non-inferiority?)
**Learning curve**	***Checklist to frame health technology assessments*** Grutters et al. (28)	What is the patient use that is relevant for the decision problem? (e.g., uptake, compliance, adherence)
	***Checklist to frame health technology assessments*** Grutters et al. (28)	What is the use of the technology by health care professionals that is relevant for the decision problem? (e.g., skills, experience, beliefs)
**Incremental innovation**	***CUA checklist*** Gerard et al. (24)	Stage of technical development of intervention
**Organizational impact**	***Checklist for economic evaluations of pharmaceuticals*** Inotai et al. (35)	Did the presentation and interpretation of the results of the economic evaluation cover every relevant aspect that needs to be considered when purchasing health care services?

CUA, cost-utility analysis.

Consequently, we expanded our search to include grey literature sources and economic evaluation methodological guidelines. We reviewed 77 sources but found no existing instruments tailored specifically for medical devices. Relevant information for only five medical device-specific features was found in six guidelines. These features included low evidence in New Zealand, learning curve effects in Japan, the UK, the Netherlands, and New Zealand, incremental innovation in France, Ireland, Japan, the Netherlands, and New Zealand, diversity in the Netherlands, and dynamic pricing in France. None of the guidelines addressed the domains of organizational impact or transferability. Additional information on medical device economic evaluations from Canada, France, Ireland, New Zealand, and the Netherlands that could not be classified under the defined medical device-specific features was also found. These countries offer recommendations such as resource measurement and costing (Canada, France, New Zealand), adverse effects (Ireland), value components beyond health outcomes (the Netherlands), and outcome measures and evaluation methods (the Netherlands). These relevant items are presented in Table 3.

**Table 3.** Medical device-specific features and relevant guideline items by country

Medical device specific feature	Country	Relevant guideline item
**Low evidence**	New Zealand (38)	Trials on medical devices may be of insufficient duration to evaluate long-term efficacy and may only report intermediate endpoints. As lower-quality evidence increases the level of uncertainty in the analysis, conservative assumptions should be applied, and extensive sensitivity analysis undertaken.
**Learning curve**	Japan (39)	Regarding the evaluation of medical devices, if there are reliable and quantitative data, analysis reflecting “learning effect” (i.e., improvement of treatment effect via the accumulation of clinicians’ experience) or “product improvement effect” can be submitted in addition to analyses not considering the effects, upon agreement in consultation with C2H.
	New Zealand (38)	In cases where there is evidence of reduced efficacy or safety in clinical practice compared with the trial, the analysis should adjust the efficacy/safety of a device in the first few years and assume increased efficacy/safety over time as operators gain experience.
	The UK (40)	Assumptions included in models should, when appropriate, be validated by a user of the technology who has experience of using it in the NHS or a user with appropriate expertise that can be applied to the technology. This is particularly relevant for the evaluation of medical devices.
	The Netherlands (41)	For many care interventions, such as medical devices, there is a learning curve that has an effect on the outcomes. This may have an impact on the external validity of the study results, such as when the findings of a study involving experienced users are generalized to the entire population. The opposite applies if outcomes of a short-term study with inexperienced users are extrapolated over a longer time horizon. It is important that the effect of any learning curve is clarified using a scenario and/or uncertainty analysis, where the learning period is excluded.
**Incremental innovation**	France (42)	The reference case analysis is time-specific. Any change in the healthcare strategy – particularly the arrival of a new comparator on the market – will invalidate the previous cost-effectiveness evaluation. The evaluation takes account of the changes in the characteristics of technologies over time (performance, cost, etc.). Where the introduction of an intervention entails the withdrawal of another intervention (e.g. new version of a medical device, industrial development strategy, etc.), the intervention replaced is to be included in the analysis to inform the decision-maker of all the consequences of the substitution.
	Ireland (43)	For medical devices, which can change substantially over time in terms of design, it must be clear that selected studies are based on the same device. Evidence of efficacy in a specific device should not be generalized to other similar devices or subsequent generations of a device unless it can be shown that they are at least equivalent and that the synthesized evidence is appropriately adjusted to account for differences.
	Japan (39)	Regarding the evaluation of medical devices, if there are reliable and quantitative data, analysis reflecting “learning effect” (i.e., improvement of treatment effect via the accumulation of clinicians’ experience) or “product improvement effect” can be submitted in addition to analyses not considering the effects, upon agreement in consultation with C2H.
	New Zealand (38)	In cases where products have been modified since the reported clinical trials, it is recommended that the assessment be based on a synthesis of the trial data (to evaluate the overall efficacy of the product group) and any further evidence available on the impact of product modifications on the efficacy of the device.
	The Netherlands (41)	For some healthcare interventions, incremental innovation throughout their life cycle is expected. This is not exceptional in the case of medical devices, but it can also apply to other interventions. The intervention could be improved further during or after an economic evaluation has been performed. As a result, it may be necessary to create a dynamic model for the effectiveness and the costs. It is important that the specifications of the care intervention investigated and any assumptions about innovation are accurately reported. The effect of any assumptions should be investigated in scenario analyses.
**Diversity**	The Netherlands (44)	The diversity of devices is enormous, and in the “healthcare market” we can distinguish two target groups of primary users of diagnostic or therapeutic devices. With regard to application on an individual level, for example, the thermometer with which diabetes patient monitor the foot temperature to prevent foot ulcers, there is not much difference with a common intervention; the market consists of the number of patients. With regards to equipment, such as a CT-scanner, the situation is less clear-cut. In that case, the first layer in the healthcare market is made up of the caregivers who will be using the device. The second layer consists of the patients on whom the caregivers will be applying the device. These two layers should be taken into account when determining the price per unit, also considering that the depreciation charges of the equipment via the procedure are influenced by the utilization degree, or in other terms, the non-optimal use of the device. That said, collective devices are often suitable for use in multiple patient populations, which may have a cost-reducing effect on the price per procedure.
**Dynamic pricing**	France (42)	Resources for which there is no tariff set (non-listed procedures, non-refundable medical devices or, heavy equipment, mobile medical units, etc.) should be valued at the average real price if observable, or via another method to be detailed. Medical devices and medicines are most often valued based on their tariff, except when this tariff does not represent all the expenses borne by the various funders: medical devices for which a reference price has been set may be marketed at a price to be freely determined by the manufacturer. Such devices are valued at the volume-weighted average price calculated on the real quantities sold. non-refundable devices sold at a price exceeding the applicable tariff are valued at the price actually paid.
**Other recommendations for medical device economic evaluations**	Canada (45)	Researchers should be careful to avoid double counting when measuring resources; for example, avoiding situations where resource use is estimated separately as well as being bundled together in a single case-mix estimate (e.g., medical devices). In such situations, researchers must be careful to avoid overestimating the use ascribed to a particular resource.
	France (42)	Micro-costing method: precise identification of the resources consumed per intervention
	Ireland (43)	All adverse effects that are of clinical or economic importance should be included in the analysis.
	New Zealand (38)	Medical devices have costs that may differ to those for medicines, and which need to be considered. These costs include, but are not limited to: one-off costs: – capital – disposal of current device(s) – costs of switching out devices already in use – implementation fixed costs: – hiring additional staff – overheads – training costs associated with use: – operating costs – maintenance and repair – consumables
	The Netherlands (41)	The primary aim of interventions in healthcare is not always to improve the health-related quality of life or extend the life expectancy of the patient. For some interventions, such as medical devices, effects may be achieved in terms of broader value components. Examples include convenience for the caregiver, or a reduction in the time required for the procedure. It may be relevant for these kinds of interventions to include those other value components in the economic evaluation as well. Methods are available that can be used to identify and quantify that valuation by directly consulting patients and users, such as a DCE and the MCDA.
	The Netherlands (41)	Due to the use of outcome measures other than the quality of life for economic evaluations of medical devices, it is not always possible to perform a CUA. Instead, a CEA is a good alternative.

C2H: Center for Outcomes Research and Economic Evaluation for Health; CT:computed tomography; CEA: cost-effectiveness analysis; CUA: cost-utility analysis; DCE: discrete choice experiment; MCDA: Multi-Criteria Decision Analysis.

## Discussion

Despite conducting a comprehensive search of peer-reviewed literature, we did not identify any methodological quality assessment instruments specifically tailored for the economic evaluation of medical devices. This gap highlights a critical area where current research is lacking. Even though Walker et al. (18) did not incorporate grey literature, to ensure comprehensiveness and avoid overlooking relevant tools, we expanded our search to include grey literature sources. This broader approach was crucial for several reasons: First, it enabled us to capture existing instruments that might not have been published in academic journals but could still be highly relevant for our review. Second, grey literature often contains supplementary information that supports or contextualizes the peer-reviewed studies (46–48). Guidelines and reports from Health Technology Assessment organizations can provide practical insights into the application and relevance of the methodological quality assessment items which is essential for developing new tools (38;40;41;43). Third, including grey literature helps mitigate publication bias, as not all valuable research is published in peer-reviewed journals (46;48). Finally, the lack of medical device-specific instruments identified through both peer-reviewed and grey literature searches underscores the need for targeted research and development in this area. Without instruments that account for the relevant aspects of medical devices – such as incremental innovation, learning curves, and dynamic pricing – it is challenging to conduct robust, high-quality economic evaluations that fully assess a device’s value over time. This gap limits policymakers’ ability to make informed decisions, potentially leading to inefficient resource allocation or delayed adoption of valuable innovations. Addressing this need is essential for establishing a framework that supports rigorous, relevant economic evaluations, ultimately enhancing healthcare quality and efficiency. Considering these gaps, we also examined the relevance of items within the assessment instruments. Remarkably, only four of the included assessment instruments contained seven relevant items, indicating a substantial gap in the comprehensiveness of tools available for the economic evaluation of medical devices. This finding underscores the need for further development of methodological quality assessment instruments that adequately capture the rigorous, relevant economic evaluations. Incorporating insights from grey literature into the development process can help ensure that new instruments are comprehensive and practically applicable.

Notably, this systematic review addresses a significant gap in the existing literature, as no prior reviews have specifically explored this research question. This systematic review adhered to rigorous standards, as outlined in the review protocol published in INPLASTY. We conducted a comprehensive literature search across multiple databases and grey literature. This approach ensured that we considered a wide range of sources and potential instruments. However, the review has limitations. Only English-language articles were included, which may introduce language bias. While prior studies, such as Morrison et al. (49), found no systematic bias from language restrictions in conventional medicine reviews, further research is needed to understand the impact of such restrictions in specialized fields like health economics. Additionally, there was no specific tool for assessing the methodological quality of included studies. Instead, we evaluated instruments based on their development, validation, applicability, previous use, citations, and updates. This approach ensured credibility, but the lack of a standardized quality assessment tool highlights another gap in literature.

## Conclusion

Existing instruments cover general items related to the conduct of health economic evaluation studies. However, there is currently a lack of a specific instrument to systematically assess the methodological quality of published economic evaluations for medical devices. To address this gap, future research should focus on developing methodological quality assessment instruments that adequately capture the complexities of medical devices.

## Supporting information

Akpinar et al. supplementary material Akpinar et al. supplementary material

## Data Availability

Data extracted from included studies will be made available upon request.

## Supplementary material

The supplementary material for this article can be found at http://doi.org/10.1017/S0266462325000212.
