# Supplementary material for: A systematic review of health economic evaluation quality assessment instruments for medical devices
Source: Int J Technol Assess Health Care. 2025 Jul 10;41(1):e40. doi: 10.1017/S0266462325000212 (PMC12257040; doi:10.1017/S0266462325000212)
Supplement: Akpinar et al. supplementary material [file S0266462325000212sup001.zip › Supplementary material 1_Search strategy.docx]

## Systematic Review Search Strategy

**Ovid MEDLINE(R) ALL <1946 to May 24, 2023>**

| # | Query | Results from 24 May 2023 |
| --- | --- | --- |
| 1 | Economics/ | 27,505 |
| 2 | Cost/ | 51,405 |
| 3 | exp Health Economics/ | 1,686,780 |
| 4 | (economic* or cost or costs or costly or costing or price or prices or pricing or pharmacoeconomic* or pharmaco-economic* or expenditure or expenditures or expense or expenses or financial or finance or finances or financed).ti,kf. | 281,308 |
| 5 | (economic* or cost or costs or costly or costing or price or prices or pricing or pharmacoeconomic* or pharmaco-economic* or expenditure or expenditures or expense or expenses or financial or finance or finances or financed).ab. /freq=2 | 380,888 |
| 6 | (cost* adj2 (effective* or utilit* or benefit* or minimi* or analy* or outcome or outcomes)).ab,kf. | 209,321 |
| 7 | checklist*.mp. | 57,762 |
| 8 | tool*.mp. | 980,172 |
| 9 | questionnaire*.mp. | 950,486 |
| 10 | CHEERS.mp. [mp=title, book title, abstract, original title, name of substance word, subject heading word, floating sub-heading word, keyword heading word, organism supplementary concept word, protocol supplementary concept word, rare disease supplementary concept word, unique identifier, synonyms, population supplementary concept word, anatomy supplementary concept word] | 515 |
| 11 | QHES.mp. [mp=title, book title, abstract, original title, name of substance word, subject heading word, floating sub-heading word, keyword heading word, organism supplementary concept word, protocol supplementary concept word, rare disease supplementary concept word, unique identifier, synonyms, population supplementary concept word, anatomy supplementary concept word] | 108 |
| 12 | PQAQ.mp. [mp=title, book title, abstract, original title, name of substance word, subject heading word, floating sub-heading word, keyword heading word, organism supplementary concept word, protocol supplementary concept word, rare disease supplementary concept word, unique identifier, synonyms, population supplementary concept word, anatomy supplementary concept word] | 8 |
| 13 | instrument*.mp. [mp=title, book title, abstract, original title, name of substance word, subject heading word, floating sub-heading word, keyword heading word, organism supplementary concept word, protocol supplementary concept word, rare disease supplementary concept word, unique identifier, synonyms, population supplementary concept word, anatomy supplementary concept word] | 1,003,431 |
| 14 | CHEC.mp. [mp=title, book title, abstract, original title, name of substance word, subject heading word, floating sub-heading word, keyword heading word, organism supplementary concept word, protocol supplementary concept word, rare disease supplementary concept word, unique identifier, synonyms, population supplementary concept word, anatomy supplementary concept word] | 193 |
| 15 | data quality.mp. [mp=title, book title, abstract, original title, name of substance word, subject heading word, floating sub-heading word, keyword heading word, organism supplementary concept word, protocol supplementary concept word, rare disease supplementary concept word, unique identifier, synonyms, population supplementary concept word, anatomy supplementary concept word] | 11,075 |
| 16 | methodological quality.mp. [mp=title, book title, abstract, original title, name of substance word, subject heading word, floating sub-heading word, keyword heading word, organism supplementary concept word, protocol supplementary concept word, rare disease supplementary concept word, unique identifier, synonyms, population supplementary concept word, anatomy supplementary concept word] | 22,595 |
| 17 | reporting quality.mp. [mp=title, book title, abstract, original title, name of substance word, subject heading word, floating sub-heading word, keyword heading word, organism supplementary concept word, protocol supplementary concept word, rare disease supplementary concept word, unique identifier, synonyms, population supplementary concept word, anatomy supplementary concept word] | 2,148 |
| 18 | assess* quality.mp. | 6,083 |
| 19 | assess* reporting quality.mp. | 42 |
| 20 | assess* data quality.mp. | 289 |
| 21 | assess* methodological quality.mp. | 1,127 |
| 22 | 1 or 2 or 3 or 4 or 5 or 6 | 2,049,969 |
| 23 | 7 or 8 or 9 or 10 or 11 or 12 or 13 or 14 | 2,777,093 |
| 24 | 15 or 16 or 17 or 18 or 19 or 20 or 21 | 41,315 |
| 25 | 22 and 23 and 24 | 2,165 |
| 26 | limit 25 to (English language and yr="2012 -Current") | 1,609 |

**Ovid EMBASE May 24, 2023**

| # | Query | Results from 24 May 2023 |
| --- | --- | --- |
| 1 | Economics/ | 244,415 |
| 2 | Cost/ | 62,490 |
| 3 | exp Health Economics/ | 1,023,971 |
| 4 | (economic* or cost or costs or costly or costing or price or prices or pricing or pharmacoeconomic* or pharmaco-economic* or expenditure or expenditures or expense or expenses or financial or finance or finances or financed).ti,kf. | 348,328 |
| 5 | (economic* or cost or costs or costly or costing or price or prices or pricing or pharmacoeconomic* or pharmaco-economic* or expenditure or expenditures or expense or expenses or financial or finance or finances or financed).ab. /freq=2 | 530,922 |
| 6 | (cost* adj2 (effective* or utilit* or benefit* or minimi* or analy* or outcome or outcomes)).ab,kf. | 292,035 |
| 7 | checklist*.mp. | 84,916 |
| 8 | tool*.mp. | 1,334,514 |
| 9 | questionnaire*.mp. | 1,259,194 |
| 10 | CHEERS.mp. [mp=title, abstract, heading word, drug trade name, original title, device manufacturer, drug manufacturer, device trade name, keyword heading word, floating subheading word, candidate term word] | 681 |
| 11 | QHES.mp. [mp=title, abstract, heading word, drug trade name, original title, device manufacturer, drug manufacturer, device trade name, keyword heading word, floating subheading word, candidate term word] | 176 |
| 12 | PQAQ.mp. [mp=title, abstract, heading word, drug trade name, original title, device manufacturer, drug manufacturer, device trade name, keyword heading word, floating subheading word, candidate term word] | 8 |
| 13 | instrument*.mp. [mp=title, abstract, heading word, drug trade name, original title, device manufacturer, drug manufacturer, device trade name, keyword heading word, floating subheading word, candidate term word] | 694,237 |
| 14 | CHEC.mp. [mp=title, abstract, heading word, drug trade name, original title, device manufacturer, drug manufacturer, device trade name, keyword heading word, floating subheading word, candidate term word] | 242 |
| 15 | data [quality.mp](http://quality.mp/). [mp=title, abstract, heading word, drug trade name, original title, device manufacturer, drug manufacturer, device trade name, keyword heading word, floating subheading word, candidate term word] | 18,136 |
| 16 | methodological [quality.mp](http://quality.mp/). [mp=title, abstract, heading word, drug trade name, original title, device manufacturer, drug manufacturer, device trade name, keyword heading word, floating subheading word, candidate term word] | 27,111 |
| 17 | reporting [quality.mp](http://quality.mp/). [mp=title, abstract, heading word, drug trade name, original title, device manufacturer, drug manufacturer, device trade name, keyword heading word, floating subheading word, candidate term word] | 2,761 |
| 18 | assess* [quality.mp](http://quality.mp/). | 9,227 |
| 19 | assess* reporting [quality.mp](http://quality.mp/). | 51 |
| 20 | assess* data [quality.mp](http://quality.mp/). | 341 |
| 21 | assess* methodological [quality.mp](http://quality.mp/). | 1,442 |
| 22 | 1 or 2 or 3 or 4 or 5 or 6 | 1,589,023 |
| 23 | 7 or 8 or 9 or 10 or 11 or 12 or 13 or 14 | 3,114,349 |
| 24 | 15 or 16 or 17 or 18 or 19 or 20 or 21 | 56,306 |
| 25 | 22 and 23 and 24 | 1,904 |
| 26 | limit 25 to (english language and yr="2012 -Current") | 1,537 |

**CINAHL EBSCOhost Research Databases May 25, 2023**

| # | Query Limiters/Expanders | Results from 24 May 2023 |
| --- | --- | --- |
| 1 | economics in healthcare | 68,832 |
| 2 | cost | 260,214 |
| 3 | cost effectiveness or cost benefit | 84,438 |
| 4 | cost utility | 7,416 |
| 5 | checklist or check list or tool | 442,320 |
| 6 | CHEERS checklist | 83 |
| 7 | QHES | 39 |
| 8 | PQAQ | 2 |
| 9 | CHEC | 68 |
| 10 | Pediatric Quality Appraisal Questionnaire | 47 |
| 11 | Consolidated Health Economic Evaluation Reporting Standards | 151 |
| 12 | Consensus Health Economic Criteria | 222 |
| 13 | Quality of Health Economic Studies | 18,780 |
| 14 | (S1 OR S2 OR S3 OR S4) | 286,030 |
| 15 | S5 AND S14 | 22,400 |
| 16 | (S5 AND S14) AND (S6 OR S7 OR S8 OR S9 OR S10 OR S11 OR S12 OR S13) | 1,905 |
| 17 | (S5 AND S14) AND (S6 OR S7 OR S8 OR S9 OR S10 OR S11 OR S12 OR S13)  Limiters - Published Date: 20120101-20231231 | 1,492 |
| 18 | (S5 AND S14) AND (S6 OR S7 OR S8 OR S9 OR S10 OR S11 OR S12 OR S13)  Limiters - English Language; Published Date: 20120101-20231231 | 1,470 |

**EconLit EBSCOhost Research Databases May 24, 2023**

| # | Query Limiters/Expanders | Results from 24 May 2023 |
| --- | --- | --- |
| 1 | economics | 1,356,130 |
| 2 | cost | 214,238 |
| 3 | health economics | 112,275 |
| 4 | cost effectiveness or cost benefit | 47,702 |
| 5 | cost utility | 13,122 |
| 6 | checklist or check list or tool | 37,832 |
| 7 | CHEERS Checklist | 12 |
| 8 | QHES | 7 |
| 9 | PQAQ | 0 |
| 10 | CHEC | 4 |
| 11 | Pediatric Quality Appraisal Questionnaire | 26 |
| 12 | Consolidated Health Economic Evaluation Reporting Standards | 17 |
| 13 | Consensus Health Economic Criteria | 45 |
| 14 | Quality of Health Economic Studies | 4,664 |
| 15 | S1 OR S2 OR S3 OR S4 OR S5 | 1,413,316 |
| 16 | (S1 OR S2 OR S3 OR S4 OR S5) AND (S7 OR S8 OR S9 OR S10 OR S11 OR S12 OR S13 OR S14) | 4,704 |
| 17 | ((S1 OR S2 OR S3 OR S4 OR S5) AND (S7 OR S8 OR S9 OR S10 OR S11 OR S12 OR S13 OR S14)) AND (S6 AND S16) | 236 |
| 18 | ((S1 OR S2 OR S3 OR S4 OR S5) AND (S7 OR S8 OR S9 OR S10 OR S11 OR S12 OR S13 OR S14)) AND (S6 AND S16) | 222 |
| 19 | ((S1 OR S2 OR S3 OR S4 OR S5) AND (S7 OR S8 OR S9 OR S10 OR S11 OR S12 OR S13 OR S14)) AND (S6 AND S16) Limiters - Published Date: 20120101-20231231 | 172 |

**Web of Science Core Collection May 25, 2023**

(((((((((((((((((((ALL=(&quot;economics&quot;)) OR ALL=(&quot;cost&quot;)) OR ALL=(&quot;health economics&quot;)) OR ALL=(&quot;cost utility&quot;)) OR ALL=(&quot;cost effectiveness&quot;)) OR

ALL=(&quot;cost benefit&quot;)) AND ALL=(&quot;checklist&quot;)) OR ALL=(&quot;tool&quot;)) OR ALL=(&quot;questionnaire&quot;)) AND ALL=(CHEERS)) OR ALL=(&quot;qhts&quot;)) OR ALL=(pmaq))

OR ALL=(&quot;check&quot;)) AND ALL=(&quot;data quality&quot;)) OR ALL=(&quot;methodological quality&quot;)) OR ALL=(&quot;reporting quality&quot;)) OR ALL=(&quot;assess* quality&quot;)) AND

ALL=(&quot;assess* reporting quality&quot;)) OR ALL=(&quot;assess* data quality&quot;)) OR ALL=(&quot;assess* methodological quality&quot;) | 1,137

**INAHTA database November 18, 2023**

searched by publication type

checklist 48

tool 430

medical device 164

guidelines 119

(((guidelines) OR (checklist) OR (tool)) AND (medical device)) AND (economic) 10

https://www.inahta.org/members/members_list/

**ISPOR webpage November 25, 2023**

Category: Pharmacoeconomic Guidelines, taskforce reports, publications

Organization: all

Country: all

Language: English only

**CADTH Grey Matters tool November 30, 2023**

Category: Heath Economics, HTA agencies

Organization: all

Country: all

Language: English only
